# Participation of Selected Soluble BMP-2 and BMP-7 Bone Morphogenetic Proteins and Their Soluble Type I ALK-1 and Type II BMPR2 Receptors in Formation and Development of Endometriosis

**DOI:** 10.3390/biomedicines9101292

**Published:** 2021-09-22

**Authors:** Joanna Janusz, Aleksandra Janusz, Zdzisława Kondera-Anasz, Justyna Sikora, Marta Smycz-Kubańska, Aleksandra Englisz, Dominika Wendlocha, Aleksandra Mielczarek-Palacz

**Affiliations:** Department of Immunology and Serology, Faculty of Pharmaceutical Science in Sosnowiec, Medical University of Silesia, 40-055 Katowice, Poland; asia25a@onet.pl (J.J.); ola.janusz@vp.pl (A.J.); zanasz@sum.edu.pl (Z.K.-A.); jsikora@sum.edu.pl (J.S.); mkubanska@sum.edu.pl (M.S.-K.); aenglisz@sum.edu.pl (A.E.); apalacz@sum.edu.pl (A.M.-P.)

**Keywords:** endometriosis, angiogenesis, BMP, ALK-1, BMPR2, ligand, receptor

## Abstract

Angiogenesis is considered to be one of the key stages in the development of endometriosis. Recent studies indicate that bone morphogenetic proteins (BMPs) and their receptors (BMPR) may play an important role in the angiogenesis process. In the literature, however, there is a lack of publications concerning binding BMPs and their receptors with the pathogenesis of endometriosis. The aim of the study was to determine the role of soluble bone morphogenetic proteins, BMP-2 and BMP-7, and their receptors, ALK-1 and BMPR2, in the process of the formation and development of endometriosis. Peritoneal fluid was collected in the proliferative phase of the menstrual cycle, from 80 women aged 21–49 years (mean age 31.3 ± 6.7 years) undergoing laparoscopy to determine the causes of primary infertility. The study involved 60 women in the I, II, III, and IV stages of the disease. The reference group consisted of 20 women who did not have endometriosis or other lesions in the pelvic area. The concentration in the peritoneal fluid of women with endometriosis was compared to the concentration of this parameter in the reference group, and a statistically significant reduction in the concentration of the BMP-2 molecule was found, as well as increasing concentrations of BMP-7, ALK-1, and BMPR2. BMP-2 and BMP-7 and their soluble receptors, ALK-1 and BMPR2, are involved in the formation of endometriosis. The changes in the concentrations of most of the tested parameters demonstrated in the study, especially in the early stages of the disease, may indicate the more effective formation of new blood vessels in this period.

## 1. Introduction

Endometriosis is a chronic inflammatory disease in which the active endometrial tissue of the uterus body occurs ectopically, outside the natural place of its location, mainly within the peritoneal cavity [1]. Irrespective of the pathomechanism of endometriosis, a necessary condition for the maintenance of endometrial tissue in an environment outside of the uterine cavity is the supply of oxygen and nutrients for ectopic endometrial cells. The ectopic endometrial tissue does not have its own blood vessel network, and is only a few millimeters in diameter; in the absence of vascularization, necrosis occurs [2]. Therefore, angiogenesis is considered to be one of the key stages in the development of endometriosis.

**Figure 1:**
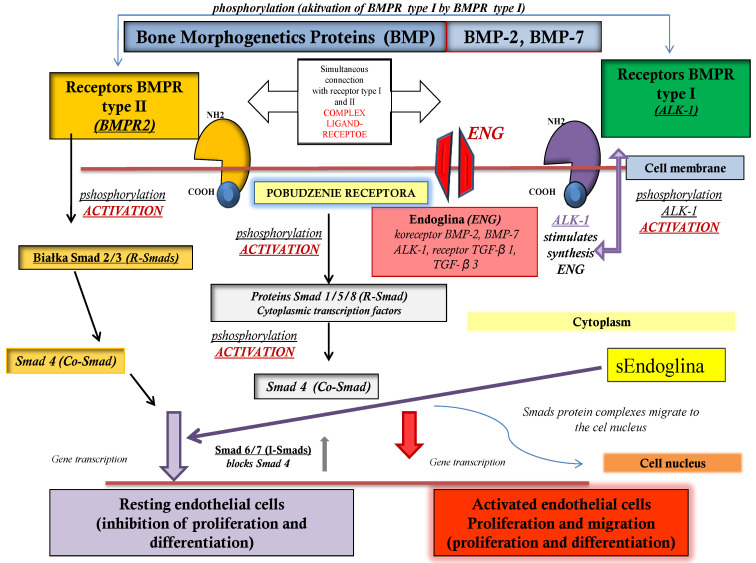
Participation of bone morphogenetic proteins and their receptors in the intercellular signaling pathway.

Important factors involved in angiogenesis, including those regulating the proliferation of vascular endothelial cells, are bone morphogenetic proteins (BMPs, Bone Morphogenetic Proteins) and their receptors (BMPRs, Bone Morphogenetics Receptors). These factors demonstrate the protective qualities of angiogenesis on endothelial cells against apoptosis, which favor their intensive proliferation and the remodeling of blood vessels, and also create conditions for the formation of a new vascular network [3]. In addition, they regulate the proliferation and differentiation of many cell types and also participate in immunological processes, which affect the migration and proliferative activity in the endothelial cells of blood vessels. This can facilitate the growth of endometrial cells and even their colonization in distant places in the body [4]. In the process of the formation and development of blood vessels, a special role is assigned to bone morphogenetic proteins, bone morphogenetic protein 2 (Bone Morphogenetic Protein 2, BMP-2) and bone morphogenetic protein 7 (Bone Morphogenetic Protein 7, BMP-7), and their receptors, type I for bone morphogenetic proteins (Activin Receptor-Like Kinase 1, ALK-1) and type II for bone morphogenetic proteins (Bone Morphogenetic Protein Receptor 2, BMPR2) [5]. Figure 1 shows the roles of bone morphogenetic proteins and their receptors in the intercellular signaling pathway.

The presence of bone morphogenetic proteins and their receptors as soluble forms makes it possible to determine their presence in biological fluids, including peritoneal fluid. Due to the fact that studies on the concentration of these parameters in peritoneal fluid among women with endometriosis have not yet been conducted, it is important to elucidate their participation in the formation and development of this disease.

### Aim

The aim of the study is to determine whether selected bone morphogenetic proteins and their soluble receptors are involved in the formation and development of endometriosis.

This goal was achieved through the recognition of the concentration value of BMP-2 and BMP-7 bone morphogenetic proteins and their receptors, type-IALK-1 and type-IIBMPR2, in the peritoneal fluid of women with endometriosis, as well as the determination of whether the tested women showed any correlation between the concentration of the examined parameters and the stage of the disease.

## 2. Materials and Methods

The study included 80 women aged 21–49 years (mean age 31.3 ± 6.7 years) undergoing laparoscopy to determine the causes of primary infertility. The examined women were patients of the Department and Clinical Ward of Gynaecology and Obstetrics, Professor K. Gibiński University Clinical Centre, Medical University of Silesia in Katowice. The inclusion criteria were as follows: written informed consent to participate in the study, regular menstrual cycles lasting 28 ± 4 days, general good health, diagnosis of primary infertility, no history of the use of hormonal substances for 3 months before laparoscopy, no history of autoimmune diseases, as well as benign or malignant lesions in the ovaries and the uterus. The exclusion criteria were as follows: failure to deliver written consent to participate in the study, irregular menstrual cycles (not lasting 28 ± 4 days), history of immunomodulators, hormonal medication, general poor health, non-malignant or malignant changes in the ovaries or the uterus. In total, 60 women aged 21–49 years (mean age 31.9 ± 7.0 years) were enrolled in the study group; among them, endometriosis in the small pelvis was diagnosed during laparoscopy and then confirmed by histopathological examination. The extent and severity of endometrial changes in the peritoneal cavity were evaluated according to the classification of the American Society of Fertility (rAFS), including the division into stages I, II, III, and IV of endometriosis. In total, 20 women were at the first stage of endometriosis, 16 women at stage II, 15 women at stage III, and 9 women were diagnosed at stage IV. The reference group consisted of 20 women aged 21–46 years (mean age, 30.4 ± 6.1 years), who had no endometriosis or other pathological changes observed in the small pelvis region during laparoscopy. All of the women were informed about the purpose of the research and they expressed their consent for the collection and use of their peritoneal fluid for scientific purposes. Approval was granted by the Bioethics Committee of the Medical University of Silesia in Katowice, No. KNW-6501-57/07, for the tests. The test material was the peritoneal fluid collected during laparoscopy from women in the proliferative phase of the menstrual cycle. Immediately after collection, the peritoneal fluid was centrifuged at 2500 rpm for 10 min at 4 °C, to separate the fluid from the cells. After centrifugation, the supernatant was divided into small portions and stored at −80 °C until the assays were made.

Peritoneal fluid concentrations were determined through the use of Enzyme Linked Immunosorbent Assays (ELISA) by Cloud-Clone Corp., Houston, state, USA for the following parameters:

- Bone morphogenetic protein 2 (Bone Morphogenetic Protein 2, BMP-2); test sensitivity: 5.5 pg/mL.
- Bone morphogenetic protein 7 (Bone Morphogenetic Protein 7, BMP-7); test sensitivity: 14.7 pg/mL.
- Soluble type I receptor for bone morphogenetic proteins (Activin Receptor Like Kinase 1, ALK1); test sensitivity: 0.049 ng/mL.
- Soluble type II receptor for bone morphogenetic proteins (Bone Morphogenetic Protein Receptor 2, BMPR2); test sensitivity: 0.117 ng/mL.

**Figure 2:**
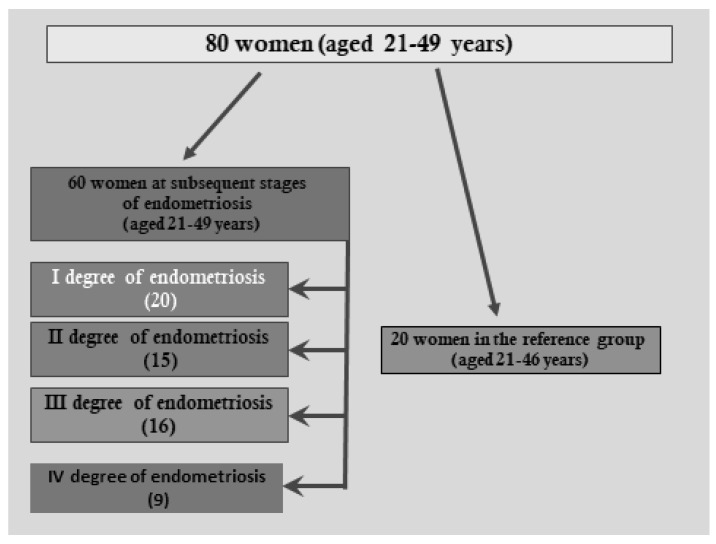
Women at subsequent stages of endometriosis and women in the reference group.

Figure 2 shows the women at subsequent stages of endometriosis and women in the reference group.

## 3. Results

### 3.1. BMP-2 Concentration

**Figure 3:**
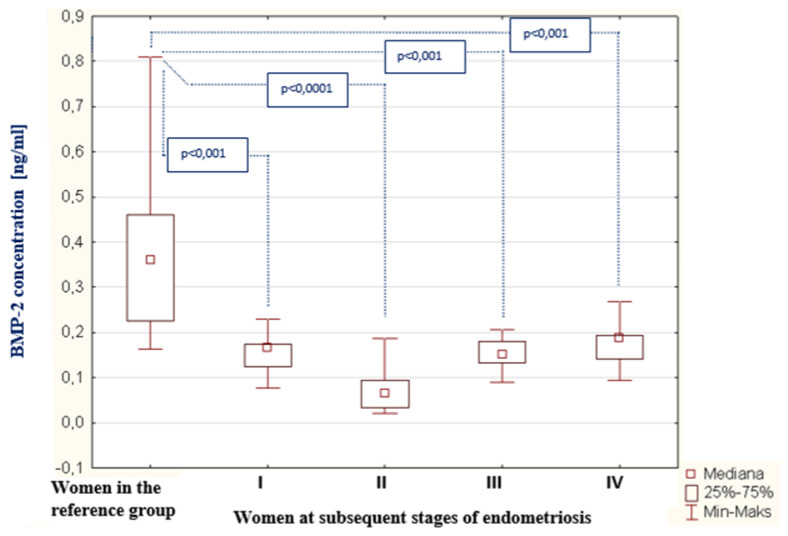
BMP-2 concentration in the peritoneal fluid of women at subsequent stages of endometriosis and women in the reference group.

In the peritoneal fluid of women with endometriosis, BMP-2 concentration decreased, in comparison with the concentration of this parameter in the reference group (*p* < 0.0001). The average BMP-2 concentration among women with endometriosis was 0.14 ng/mL (Q1: 0.09 and Q3: 0.18), and in the reference group: 0.36 ng/mL (Q1: 0.46 and Q3: 1.66). The concentration of BMP-2 in the peritoneal fluid of women with subsequent degrees of endometriosis and in the reference group is shown in Figure 3. The analysis of the results demonstrated a statistically significant decrease in the BMP-2 concentration in the fluid of women at stage II of endometriosis, compared to the concentration of this parameter among women at stage I (*p* < 0.05). In the group of women with stage III endometriosis, the concentration of BMP-2 demonstrated a statistically significant increase compared to the concentration of this parameter in the fluid of women at stage II (*p* < 0.05). In the fourth stage of endometriosis, the BMP-2 concentration reached the highest value; however, compared to the concentration of this parameter in the peritoneal fluid of women at the third stage of endometriosis, this was not a statistically significant change. In the 4th stage of endometriosis, the BMP-2 concentration reached the highest value, however, comparing to the concentration of this parameter in the peritoneal fluid of women with the 3rd degree of endometriosis, this was not a statistically significant change. There was no statistically significant correlation between BMP-2 concentration and the degree of endometriosis.

### 3.2. BMP-7 Concentration

**Figure 4:**
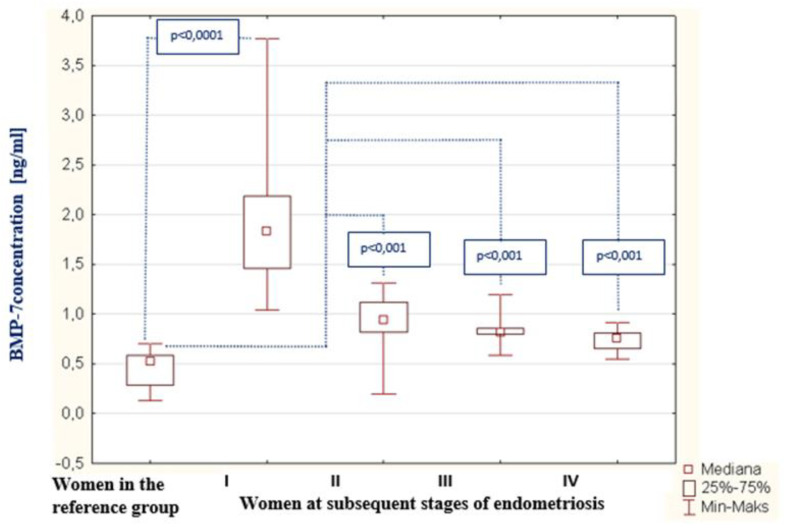
BMP-7 concentration in the peritoneal fluid of women at subsequent stages of endometriosis and women in the reference group.

**Figure 5:**
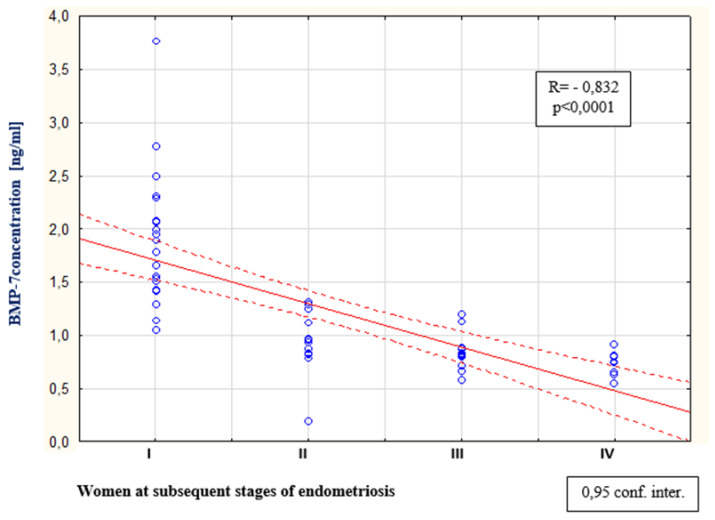
Linear regression curve illustrating the correlation between the concentration of BMP-7 in the peritoneal fluid of women with endometriosis and the subsequent stages of the disease.

As a result of the tests carried out in the peritoneal fluid of women with endometriosis, a statistically significant increase in BMP-7 concentration was found compared to the concentration of this protein among women from the reference group (*p* < 0.0001). The average concentration of BMP-7 among women with endometriosis was 0.93 ng/mL (Q1: 0.81 and Q3: 1.46), and in the reference group, this was 0.52 ng/mL (Q1: 0.28 and Q3: 0.59). The concentrations of BMP-7 in the peritoneal fluid of women with subsequent degrees of endometriosis, and in the reference group, are shown in Figure 4. The analysis of the results showed a statistically significant decrease in BMP-7 concentration in the second stage of endometriosis compared to the concentration of this protein among women with the first stage of the disease (*p* < 0.0001). A statistically insignificant decrease in BMP-7 concentration was found among women with stage III endometriosis compared to the level of this parameter in the fluid of women at stage II of the disease. A statistically insignificant decrease in BMP-7 was demonstrated among women at stage IV of endometriosis compared to the concentration of this parameter among women at stage III of the disease and a statistically insignificant decrease in BMP-7 was demonstrated among women with stage I of endometriosis comparing to the concentration of this parameter among women with stage IV of the disease. It was observed that, in the fluid of women with stage IV endometriosis, the concentration of BMP-7 was the lowest in comparison to the concentration of this protein among women at stages III, II and I of the disease. A negative and statistically significant relationship between the concentration of BMP-7 in the peritoneal fluid and the stage of endometriosis (*p* < 0.0001; R = −0.832) was also proven, which is shown in Figure 5. The analysis of the results did not show a statistically significant relationship between the concentration of bone morphogenetic proteins -2 and -7 in the peritoneal fluid of women with endometriosis.

### 3.3. ALK-1 Concentration

**Figure 6:**
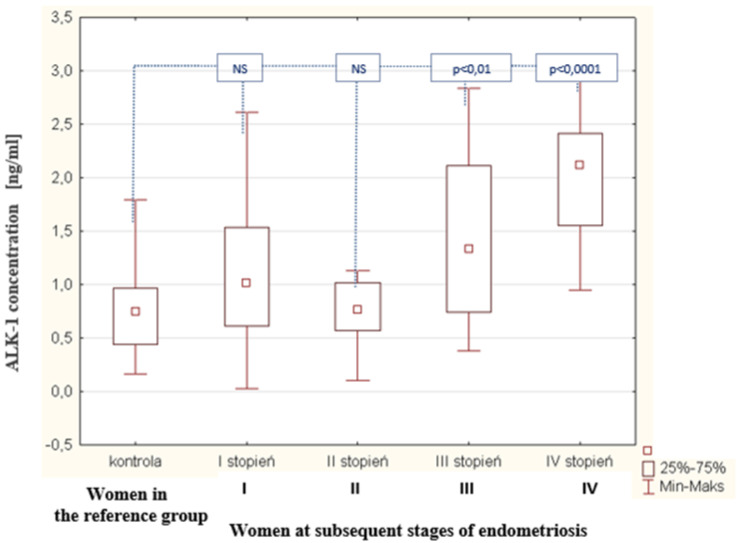
ALK-1 concentration in the peritoneal fluid of women at subsequent stages of endometriosis and women in the reference group.

**Figure 7:**
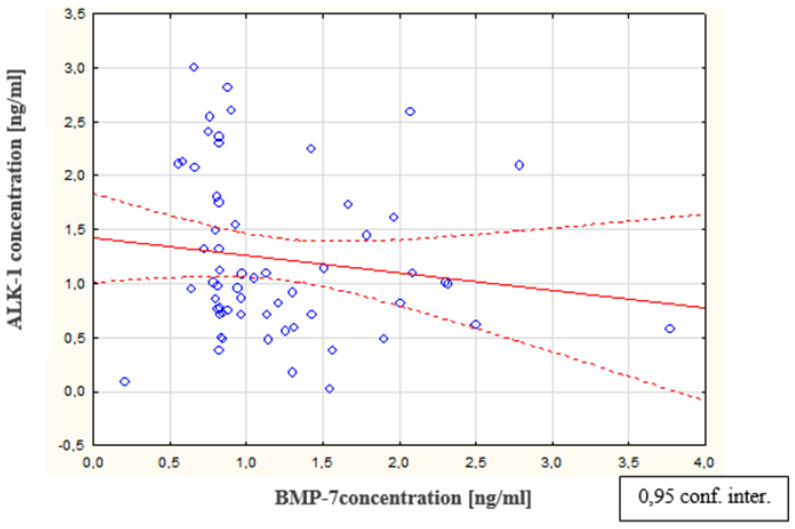
Linear regression curve illustrating the correlation between concentrations of ALK-1 and BMP-7 in the peritoneal fluid of women with endometriosis.

As a result of the tests carried out in the peritoneal fluid of women with endometriosis, a statistically significant increase was observed in the concentration of ALK-1 in the peritoneal fluid of women with endometriosis, compared to the concentration of this parameter in the reference group (*p* < 0.0001). The average concentration of ALK-1 in women with endometriosis was 1.01 ng/mL (Q1: 0.72 and Q3: 1.75); in the reference group, this was 0.75 ng/mL (Q1: 0.44 and Q3: 0.96). The concentrations of ALK-1 in the peritoneal fluid of women with subsequent degrees of endometriosis and in the reference group are shown in Figure 6. The analysis of the results demonstrated a statistically significant decrease in BMP-7 concentration at the second stage of endometriosis, compared to the concentration of this protein in women at the first stage of the disease (*p* < 0.0001). A statistically insignificant decrease in BMP-7 concentration was found in women at stage III endometriosis compared to the level of this parameter in the fluid of women at stage II of the disease. A statistically insignificant decrease in BMP-7 concentration was also observed in women at stage IV endometriosis compared to the concentration of this parameter in women at stage III of the disease. It was observed that, in the fluid of women with stage IV endometriosis, the concentration of BMP-7 was the lowest, compared to the concentration of this protein in women at stages III, II, and I of the disease. In addition, it was shown that there is no statistically significant relationship between the concentration of the ALK-1 receptor in peritoneal fluid and the concentration of bone-2 morphogenetic protein in the peritoneal fluid of women with endometriosis. In contrast, a negative statistically significant relationship was observed between the concentrations of ALK-1 receptor in peritoneal fluid and bone morphogenetic protein-7 in women with endometriosis (*p* < 0.01; R = −0.254). A linear regression curve illustrating this relationship is shown in Figure 7.

### 3.4. BMPR2 Concentration

**Figure 8:**
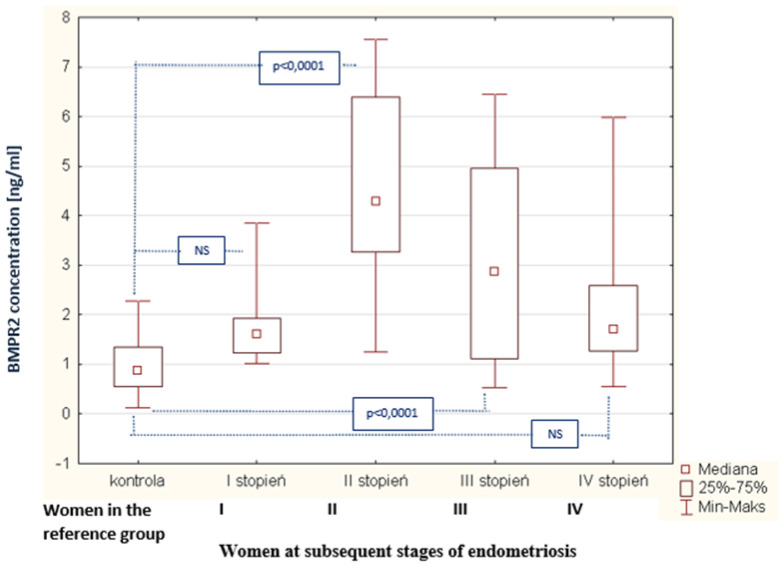
BMPR2 concentration in the peritoneal fluid of women at subsequent stages of endometriosis and women in the reference group.

**Figure 9:**
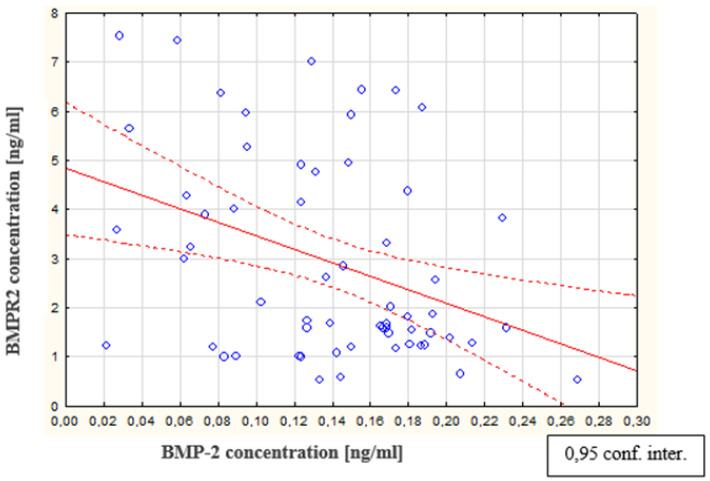
Linear regression curve illustrating the correlation between the concentrations of BMPR2 and BMP-2 in the peritoneal fluid of women with endometriosis.

As a result of the tests performed in the peritoneal fluid of women with endometriosis, a statistically significant increase in BMPR2 concentration in the peritoneal fluid of women with endometriosis was found, compared to the concentration of this parameter in the reference group (*p* < 0.0001). The average concentration of BMPR2 in women with endometriosis was 1.87 ng/mL (Q1: 1.27 and Q3: 4.34), and in the reference group, this was 0.87 ng/mL (Q1: 0.55 and Q3: 1.36). The concentrations of BMPR2 in the peritoneal fluid of women with subsequent degrees of endometriosis and in the reference group are shown in Figure 8. The analysis of the results demonstrates a statistically significant increase in BMPR2 concentration in the fluid of women at stage II of endometriosis, compared to the concentration of this receptor in women at stage I (*p* < 0.0001). It is worth emphasizing that, in women at the first stage of endometriosis, the BMPR2 concentration was the lowest while, in those at the second stage of the disease, the level of the examined receptor reached the highest value. It was also observed that, at the third stage of endometriosis, the concentration of BMPR2 demonstrated a statistically significant decrease compared to the concentration in the fluid of women at the second stage of the disease (*p* <0.0001). However, in women at the fourth stage of endometriosis, the concentration of the studied parameter decreased compared to the concentration in those at the third stage of endometriosis, and this was not a statistically significant change. It was also shown that there is no statistically significant relationship between the concentration of the BMPR2 receptor and the concentration of bone morphogenetic protein 7 in the peritoneal fluid of women with endometriosis. In addition, no statistically significant correlation was found between the type-II receptor concentration for BMPR2 and the type I receptor concentration for ALK-1. In contrast, a negative statistically significant relationship between BMPR2 receptor concentration and bone morphogenetic protein 2 concentration (*p* < 0.01; R = −0.291) is shown in Figure 9.

## 4. Discussion

Current research has shown that, in the ectopic endometrium of women with endometriosis, there is a disorder of intercellular transmission, which involves the BMP-2 signaling molecule. This protein is a signaling pleiotropic molecule and is able to regulate proliferation and differentiate many cell types. Among them are pericytes, which are important structural and functional elements in blood vessels. These cells are responsible for strengthening the structure of the basement membrane of new blood vessels. In contrast, pathological conditions favor the process of tissue fibrosis [6,7]. The reduced concentration of BMP-2 in the peritoneal fluid of women with endometriosis, demonstrated in this work the process of pericyte proliferation, thus disrupting the normal structure of blood vessels, which can promote endometrial fibrillation and the formation of adhesions. Similar studies were conducted by Matsuzaki et al. [8], who showed that, within the ectopic endometrium, blood vessels are formed, which shows functional and anatomical immaturity. These vessels contain a reduced number of pericytes, which causes the destabilization of their structure, manifesting in numerous gaps in the basement membrane and in the endothelial layer. As a result, blood vessels with abnormal functions are formed. It can also be assumed that the reduced BMP-2 concentration in women with endometriosis indirectly affects the number of peritoneal macrophages and their activity, which allows ectopic endometrial cells to implant, vascularize and survive outside of the uterine cavity. The lower BMP-2 concentration in the peritoneal fluid may also adversely affect the decidualization process in women with endometriosis, leading to problems with conception and pregnancy. Studies by Large et al. [9] have shown that infertility, preeclampsia, and miscarriages can be the result of a decrease in the expression of the gene encoding the BMP-2 protein in endometrial cells. This may promote endometrial dysfunction and, thus, hinder pregnancy and miscarriages. Current research indicates the participation of the BMP-7 molecule in the pathomechanism of heavy menstrual bleeding. Meanwhile, one of the clinical symptoms of endometriosis is menstrual disorders, characterized by heavy bleeding lasting for over 8 days, accompanied by severe pain [10]. Richards et al. [11] have shown that women with heavy menstrual bleeding have increased expression of the gene encoding the BMP-7 molecule. According to the authors, this may indicate the role of this protein in regulating menstrual bleeding. It is probably due to the influence of BMP-7 on the intensity of bleeding that this molecule may promote the formation of endometrial implants. Increased concentration of BMP-7 in the peritoneal fluid of sick women may suggest that this molecule participates in the regulation of menstrual bleeding, and their prolongation promotes the implantation of endometrial cells from retrograde heavy bleeding. This may explain the increase in the concentration of BMP-7 in women with stage I endometriosis who do not develop adhesions and significant reduction of the active surface of the endometrium, which occurs in stage IV of the disease. In conjunction with the theory of retrograde menstrual and immune theory, disturbances in the body’s distribution of bone morphogenetic proteins may perhaps explain why not all women who develop retrograde menstruation develop endometriosis. The BMP-7 molecule has pleiotropic effects [12]. Kodama et al. [13], based on the assessment of the expression of the gene encoding the BMP-7 molecule, showed that this protein inhibits the proliferation of uterine endometrial cells and is an important regulator of the decidualization process. Monsivais et al. came to similar conclusions [14], and suggest that the BMP-7 molecule is an important factor in the blastocyst implantation process, contributing to normal embryonic development. Analyzing the results of this study, it can be assumed that the increased concentration of BMP-7, demonstrated in the peritoneal fluid of women with endometriosis, may disturb decidualization, which can adversely affect the fertility of women with endometriosis. This may be due not only to the appearance of fibrous changes and adhesions associated with endometriosis, but could also be the consequence of the impaired release of the BMP-7 molecule into the peritoneal fluid and the associated change in the expression of this protein on the surface of endometrial cells. Bone morphogenetic proteins are signaling molecules whose cellular effect is the result of a reaction between the BMP ligand, BMPR type-I and -II membrane receptors, and the helper receptor. It is thought that both BMP molecules and their receptors are important factors in the regulation of the angiogenesis process. In addition, by activating the appropriate signaling pathways, they can affect apoptosis processes and the adhesion of vascular endothelial cells. Therefore, when there is an imbalance between ligand and receptor concentrations, they may play a role in the emergence and development of diseases [15]. In the available literature, there is no information regarding the role of soluble BMP type-I and -II receptors in the pathogenesis of endometriosis. ALK-1 was the first receptor analyzed in this work. ALK-1 is a receptor for type-I bone morphogenetic proteins, mainly located on endothelial cells of blood vessels and associated with the angiogenesis process. As a result of the conducted studies, an increase in ALK-1 concentration in the peritoneal fluid of women with endometriosis was found, compared to the concentration of this receptor in the reference group. A statistically significant negative correlation between ALK-1 receptor concentration and BMP-7 ligand concentration was also found. The study shows that, in women with endometriosis, along with the increase in the peritoneal fluid concentration of the ALK-1 receptor, a decrease in the concentration of the BMP-7 ligand is observed. It was also observed that the increased concentration of ALK-1 in the peritoneal fluid of women with endometriosis may disturb the activation of the pathway, with a ligand and a receptor present on the surfaces of vascular endothelial cells and endometrial cells. Consequently, this may promote the incorrect release of the soluble form of this receptor into the peritoneal fluid. Increased concentration of this receptor may also result from its reduced binding to the BMP ligand on the surface of vascular endothelial cells and the endometrium, or may be the result of the increased release of its soluble form into the peritoneal fluid of women with endometriosis. In the available literature, there are no reports relating ALK-1 to the etiopathogenesis of endometriosis. However, it is noted that this receptor plays an important role in the formation of new blood vessels [12]. The results of the study demonstrate an observed increase in the concentration of the ALK-1 receptor in the peritoneal fluid of women with endometriosis. Perhaps women with endometriosis have a disorder concerning the binding of this receptor to the BMP ligand on the surface of vascular endothelial cells, resulting in increased levels of its soluble form. As a consequence, this may interfere with the maturation process of blood vessels and promote the formation of vessels with abnormal structure and function. This is confirmed by the results of the study by Matsuzaki et al. [8], who, in histopathological preparations from women with endometriosis, observed the presence of blood vessels with an abnormal structure. In turn, Goumans et al. [16] showed that the intercellular signaling pathway, involving TGF-β/ALK1, is important in the migration and proliferation of endothelial cells occurring during angiogenesis. It can, therefore, be assumed that the increased concentration of ALK-1 in peritoneal fluid suggests that, in women with moderate endometriosis compared to women with advanced disease, the process of migration and the proliferation of vascular endothelial cells may increase. As a result, this may favor the formation and development of endometrial changes in distant parts of the body. Therefore, the increased concentration of ALK-1 in the peritoneal fluid of women with endometriosis may indicate the participation of this receptor in the disruption of the formation and maturation processes of blood vessels in the ectopic endometrium. The disturbed distribution of the ALK-1 receptor into the peritoneal fluid may promote endometriosis disorders in the maturation of blood vessels in the ectopic endometrium. The next parameter evaluated in the work was BMPR2. This is a receptor for type-II bone morphogenetic proteins. It was found that, in the peritoneal fluid of women with endometriosis, there was an increased level of BMPR2 compared to the concentration of this receptor in women from the reference group. As a result of the analysis, it was also shown that, among women with endometriosis, there is a statistically significant negative correlation between the BMPR2 receptor and the concentration of the soluble BMP-2 molecule. It has been shown that, as the concentration of the soluble type-II receptor, BMPR2, increases, the concentration of the BMP-2 ligand decreases. However, in the peritoneal fluid of women with endometriosis, no statistically significant correlation was found between the concentration of the BMPR2 receptor and the concentration of the BMP-7 signaling molecule. It seems likely that the observed reduced BMP-2 ligand concentration is due to an increase in soluble BMPR2 receptor concentration. As a result, this may affect the activation of intercellular pathways involving BMPR2, which are located on the cell surface of the ectopic endometrium and the ligand present in the peritoneal fluid in soluble form. The results of this study suggest that increased concentration levels of BMPR2 in the peritoneal fluid of women with endometriosis may affect the intercellular transmission, involving the ligand and receptor for BMP. This may result in the abnormal release of the soluble form of this receptor into the peritoneal fluid. The demonstrated increased concentration of soluble BMPR2 may be due to reduced binding of this receptor with the BMP ligand on the surface of vascular endothelial cells and endometrium, or also from its increased release into the peritoneal fluid. The obtained results may indicate that the increased BMPR2 concentration in women with endometriosis results from the increased release of the soluble form of this receptor into the peritoneal fluid, and not from the reduced binding of BMPR-2 with the BMP-2 ligand on the surface of the ectopic endometrium cells. According to Kirsch et al. [17], the BMPR2 receptor has a high affinity for BMP-2 and BMP-7 ligands. However, ligand binding to receptors only occurs if BMPR2 has previously been bound to a BMP type-I receptor that is required to enhance ligand binding. A statistically significant negative correlation between BMPR2 and BMP-2 concentrations, demonstrated through the absence of a statistically significant correlation between the concentrations of this receptor and BMP-7, may suggest that the BMPR2 receptor has a greater affinity for the BMP-2 molecule than BMP-7. Lee et al. [18] observed that bone morphogenetic protein receptors present on the surface of cells, before binding to the soluble form of the ligand, occur in the form of homomeric, as well as heteromeric, complexes and have low expression. However, upon ligand attachment, most BMP receptors form heteromeric complexes, and the expression of these receptors significantly increases. The statistically significant negative correlation between the concentration of the soluble BMPR2 receptor and the concentration of the soluble BMP-2 molecule, demonstrated in the work, may be the effect of the BMPR2 receptor binding with the BMP-2 ligand. In addition, increased BMPR2 concentration in the peritoneal fluid of women with endometriosis may indirectly indicate that this receptor is present on cells in the form of a heteromeric complex bound to the soluble ligand. As a result, this increases soluble BMPR2 concentration and a decrease in BMP-2 concentration in the peritoneal fluid of women with endometriosis. This is consistent with the results obtained in this work, which observed a reduced concentration of BMP-2 in the peritoneal fluid of women with endometriosis. The results of the conducted research indicate that one of the factors contributing to the survival of ectopic endometrial cells outside of their physiological location may be disorders occurring in women with endometriosis, which are associated with the release of BMPR2 into the peritoneal fluid. The changes in the concentration of the examined bone morphogenetic proteins and their soluble receptors in the peritoneal fluid of women with endometriosis, shown especially in the early stage of the disease, may indicate an increase in the formation of new blood vessels during this period. In addition, the changes in the concentration of the parameters studied in the peritoneal fluid of women with endometriosis in subsequent stages of the disease may favor the angiogenesis process, thanks to which endometrial cells inhabit various places in the body. As a result, this may promote the protection of the endothelial cells in blood vessels that are present in the endometrium against apoptosis, as well as promoting their proliferation, thereby enabling the spread of endometrial cells by blood vessels and the development of endometrial changes.

## 5. Conclusions

Changes in the concentration of bone morphogenetic proteins, BMP-2 and BMP-7, and their soluble type-I receptors, demonstrated in the peritoneal fluid of women with endometriosis, ALK-1, and type-IIBMPR, indicate their participation in the pathogenesis of endometriosis, mainly by affecting the process of angiogenesis. In addition, disturbances in the distribution of BMP-2 and BMP-7 molecules can be an important factor in the process of decidualization in women with endometriosis. As a result, this can lead to problems with conceiving and miscarriages for these women.

## Data Availability

The data used in this study are available in this article.
